# *bla*_KPC-2_ overexpression and *bla*_GES-5_ carriage as major imipenem/relebactam resistance mechanisms in *Pseudomonas aeruginosa* high-risk clones ST463 and ST235, respectively, in China

**DOI:** 10.1128/aac.00675-23

**Published:** 2023-10-11

**Authors:** Yue Li, Li Fang, Mengqian Dong, Heng Cai, Xiaoting Hua, Yan Jiang, Yunsong Yu, Qing Yang

**Affiliations:** 1 Department of Infectious Diseases, Sir Run Run Shaw Hospital, Zhejiang University School of Medicine, Hangzhou, China; 2 Regional Medical Center for National Institute of Respiratory Diseases, Sir Run Run Shaw Hospital, Zhejiang University School of Medicine, Hangzhou, China; 3 Key Laboratory of Microbial Technology and Bioinformatics of Zhejiang Province, Hangzhou, China; 4 Department of Laboratory Medicine, The First Affiliated Hospital, Zhejiang University School of Medicine, Hangzhou, China; 5 State Key Laboratory for Diagnosis and Treatment of Infectious Diseases, National Clinical Research Center for Infectious Diseases, Collaborative Innovation Center for Diagnosis and Treatment of Infectious Diseases, The First Affiliated Hospital, Zhejiang University School of Medicine, Hangzhou, China; Columbia University Irving Medical Center, New York, New York, USA

**Keywords:** *bla*_KPC-2_, *bla*_GES-5_, imipenem/relebactam, high-risk clone, carbapenem-resistant *Pseudomonas aeruginosa*, ST235, ST463

## Abstract

*Pseudomonas aeruginosa* high-risk clones pose severe threats to public health. Here, we characterize the imipenem/relebactam (IR) resistance mechanisms in *P. aeruginosa* high-risk clones sequence type 235 (ST235) and ST463 in China. Minimum inhibitory concentrations (MICs) were determined, and Illumina short-read sequencing was performed for 1,168 clinical carbapenem-resistant *P. aeruginosa* (CRPA) isolates. The gene copy number and expression level were analyzed by Illumina sequencing depth and reverse transcription-quantitative PCR, respectively. Resistance conferred by *bla*_GES-5_ was evaluated by cloning experiments. ST463 and ST235 accounted for 9.8% (115/1,168) and 4.5% (53/1,168) of total isolates, respectively, and showed high frequencies of extensively drug-resistant and difficult-to-treat resistant phenotypes. The overall IR-resistant rate in CRPA was 21.0% (245/1,168). However, the IR resistance rate was 81.7% (94/115) in ST463-PA and 52.8% (28/53) in ST235-PA. Of the ST463 isolates, 92.2% (106/115) were *Klebsiella pneumoniae* carbapenemase-producing *P. aeruginosa* (KPC-PA), and all 94 IR-resistant ST463-PA produced KPC-2. Compared to IR-susceptible ST463 KPC-2-PA, IR-resistant ST463 KPC-2-PA exhibited significantly higher *bla*_KPC-2_ copy numbers and expression levels. In ST463 KPC-2-PA, 16 mg/L relebactam resulted in additional fourfold reductions in imipenem MIC_50/90_ values compared to 4 mg/L relebactam. In ST235, 1.9% (1/53) carried *bla*_IMP_ carbapenemase and 54.7% (29/53) carried *bla*_GES_ carbapenemase. Other than the IMP producer, all 27 IR-resistant ST235-PA produced GES-5. Cloning experiments revealed that imipenem resistance in *bla*_GES-5_-carrying PAO1 transformants was generally unaffected by relebactam. In conclusion, IR-resistant CRPA isolates in China were mainly distributed in *P. aeruginosa* high-risk clones ST463 and ST235. The major underlying IR resistance mechanisms were *bla*_KPC-2_ overexpression and *bla*_GES-5_ carriage.

## INTRODUCTION

Widely present in clinical settings, carbapenem-resistant *Pseudomonas aeruginosa* (CRPA) has emerged as an urgent public health issue. Although *P. aeruginosa* isolates usually exhibit great clonal diversity, the dissemination of certain high-risk clones is an essential step for CRPA to maintain an antibiotic-resistant phenotype. Among the high-risk clones detected globally, sequence type 235 (ST235) has been identified across five continents and is considered the most widespread (1). Remarkably, in CRPA isolates from China, ST235 has been outweighed by a recently reported epidemic clone, ST463 (2). A consensus has been reached that ST463 is an emerging high-risk clone worth noting.

Relative to other CRPA infections, a substantial increase in mortality has been demonstrated for ST235 and ST463 CRPA infections, especially in patients with bloodstream infections (BSIs) (3 – 5). The higher mortality rates observed could be largely explained by two common clonal characteristics of ST235 and ST463. First, because of horizontally acquired and mutation-driven resistance, ST235-PA and ST463-PA often exhibit a multidrug-resistant (MDR), extensively drug-resistant (XDR), or pandrug-resistant (PDR) profile (6, 7), which limits treatment choices and could lead to inappropriate empirical antibiotic therapy. Second, ST235-PA and ST463-PA have been reported to harbor a large number of virulence determinants, thus leading to more severe infections (6, 8). Considering the limited activity of traditional antipseudomonals and the pathogenicity of ST235 and ST463 isolates, evaluations of new antimicrobial activity are urgently needed.

Imipenem/relebactam (IR), a novel β-lactam-β-lactamase inhibitor (BLBLI) combination, has been developed and applied clinically to treat CRPA infections. Relebactam exerts strong inhibitory effects on many class A and class C β-lactamases and is almost unaffected by OprD inactivation or efflux pump extrusion (9, 10). Consequently, IR has shown considerable antibacterial activity against *P. aeruginosa*. In a multicenter study involving 4,927 MDR-PA isolates from adult patients, although 13.5% of samples produced metallo-β-lactamases (MBLs), nearly 70% of isolates were still susceptible to IR (11).

Currently, although many studies emphasize the *in vitro* activity of IR against *P. aeruginosa*, the IR resistance mechanisms in CRPA high-risk clones remain largely unexplored. In this study, we evaluated the IR resistance profiles in clinical CRPA isolates from China, with a particular focus on the IR activity against ST235 and ST463 CRPA strains. Notably, we identified a surprisingly high IR resistance rate in ST235 and ST463 CRPA isolates. The underlying IR resistance mechanisms were further explored in detail.

## MATERIALS AND METHODS

### Bacterial isolates and antimicrobial susceptibility testing

A total of 1,168 clinical CRPA isolates, defined as resistant to either meropenem or imipenem according to the Clinical and Laboratory Standards Institute (CLSI) breakpoints (12), were included in this study. These isolates were obtained from two separate epidemiological survey programs. In the first survey conducted in a tertiary hospital in Hangzhou, China, 839 nonduplicated CRPA strains isolated from various specimens were collected during 2011–2020. In the second survey of patients diagnosed with hospital-acquired pneumonia from 33 tertiary hospitals in China, 329 nonduplicated CRPA strains isolated from sputum or bronchoalveolar lavage fluid were collected during 2019–2020. A nonduplicated CRPA strain was defined as one CRPA strain per patient during a single hospitalization.

For all 1,168 CRPA isolates, the minimum inhibitory concentrations (MICs) of amikacin, imipenem, meropenem, IR (relebactam fixed at 4 mg/L), ceftazidime, cefepime, ciprofloxacin, levofloxacin, piperacillin/tazobactam, aztreonam, fosfomycin, and polymyxin B were determined using the broth microdilution method with Thermo Scientific Sensititre System. To facilitate comparison between different antimicrobials in ST463 KPC-2-producing and ST235 GES-5-producing strains, the MICs of imipenem and IR (relebactam fixed at 4 mg/L or 16 mg/L) were simultaneously obtained by the agar dilution method described in CLSI standard M07 (13). *Escherichia coli* ATCC 25922, *P. aeruginosa* ATCC 27853, and *Klebsiella pneumoniae* ATCC 700603 and ATCC BAA-1705 were used as quality controls. For all antimicrobials except fosfomycin, CLSI breakpoints (12) for *P. aeruginosa* were adopted for result interpretation. For fosfomycin, CLSI breakpoints (12) for *Enterobacterales* were adopted. The MDR/XDR/PDR phenotypes and the difficult-to-treat resistant (DTR) phenotype were defined according to the criteria established by Magiorakos et al. and Tamma et al. respectively (14, 15).

### Whole-genome sequencing (WGS) and gene copy number calculation

The genomic DNA of all CRPA isolates was extracted using QIAamp DNA Mini Kit (Qiagen, Hilden, Germany) and underwent short-read sequencing (Illumina, San Diego, USA). *De novo* assembly was accomplished using shovill version 0.9.0 (https://github.com/tseemann/shovill). Multilocus sequence typing (MLST) was performed, and antimicrobial resistance genes were detected using mlst version 2.19.0 (https://github.com/tseemann/mlst) and ABRicate version 1.0.0 (https://github.com/tseemann/abricate). The *oprD* gene of clinical strains was compared with that of *P. aeruginosa* PAO1. OprD inactivation was confirmed if any of the following criteria were met: (1) truncation or absence of the *oprD* gene; (2) mutations leading to an OprD frameshift; and (3) mutations leading to the creation of a premature stop codon in OprD.

For KPC-2-producing strains, the *bla*_KPC-2_ copy number was further calculated based on WGS. For each sample, a SAM alignment file was obtained by mapping Illumina raw reads to references (*bla*_KPC-2_ gene and seven MLST alleles) using bowtie2 version 2.2.5 (https://github.com/BenLangmead/bowtie2) with the option “-I 0 -X 800 --fast -q.” After transforming the alignment results into BAM files, the sequencing depths of the *bla*_KPC-2_ gene and seven MLST alleles were analyzed by BEDTools version 2.30.0 (https://github.com/arq5x/bedtools2) with the option “bedtools coverage -mean.” Finally, the *bla*_KPC-2_ copy number of each strain was calculated as the depth of *bla*_KPC-2_ divided by the average depth of the seven MLST alleles.

### Core gene alignment and phylogenetic tree construction

For IR-resistant *P. aeruginosa* isolates, genome assemblies were first annotated using Prokka version 1.14.6 (https://github.com/tseemann/prokka). Core gene alignment was then obtained by pangenome analysis using Panaroo version 1.2.9 (16). The phylogenetic tree was finally constructed using IQ-TREE version 2.1.4 (17) and visualized using iTOL version 6.8 (18).

### Quantitative determination of gene expression level

To compare the expression levels of *bla*_KPC-2_, *mexA*, *mexD*, *mexE,* and *mexY* genes in clinical isolates with different resistance phenotypes, we extracted the total RNA of selected strains using Total RNA Kit I (Omega Bio-Tek, Georgia, USA). Reverse transcription was conducted using PrimeScript RT Reagent Kit (Takara, Beijing, China). Quantitative PCR was performed in biological triplicates using TB Green Premix ExTaq (TaKaRa, Beijing, China). Each biological triplicate was assayed in technological triplicates. The housekeeping gene *rpoD* was chosen as the endogenous reference for normalization. The primers used for quantitative PCR are listed in Table S1.

### Cloning experiments

The *bla*_GES-5_ and *bla*_GES-15_ genes with the same upstream promoter sequences were amplified from clinical strains R16-15 and R20-99, respectively. The cloned sequences were homologously recombined into the pGK1900 plasmid. The constructed plasmids were then transformed into both *E. coli* DH5α and *P. aeruginosa* PAO1. Transformants verified by Sanger sequencing were eventually selected for antimicrobial susceptibility testing. The primers used for cloning experiments are listed in Table S1.

### Statistical analysis

Statistical analysis was performed using the Fisher’s exact test, the Mann‒Whitney U test, and the Kruskal‒Wallis test in SPSS version 25. The results were considered statistically significant when *P* < 0.05.

## RESULTS

### IR resistance profiles of clinical CRPA isolates with different STs

Among the 1,168 CRPA isolates included in this study, MLST analysis revealed 285 different STs (excluding 69 isolates belonging to unknown STs). In 21 out of these 285 STs, the CRPA strain numbers were more than nine (Fig. S1). ST463 (115/1,168, 9.8%) and ST235 (53/1,168, 4.5%) were found to be the most prevalent STs. The resistance phenotypes of ST463-PA and ST235-PA to traditional antipseudomonal agents were compared with those of *P. aeruginosa* from other STs. The frequency of XDR/PDR phenotype was 88.7% (102/115) for ST463-PA and 73.6% (39/53) for ST235-PA (Fig. S2a); the frequency of DTR phenotype was 90.4% (104/115) for ST463-PA and 52.8% (28/53) for ST235-PA (Fig. S2b). In contrast, the proportions of XDR/PDR-PA and DTR-PA in CRPA isolates from other STs were only 24.4% (244/1,000) and 24.5% (245/1,000), respectively. Therefore, the resistance status of ST463-PA and ST235-PA was significantly more severe.

**Fig 1:**
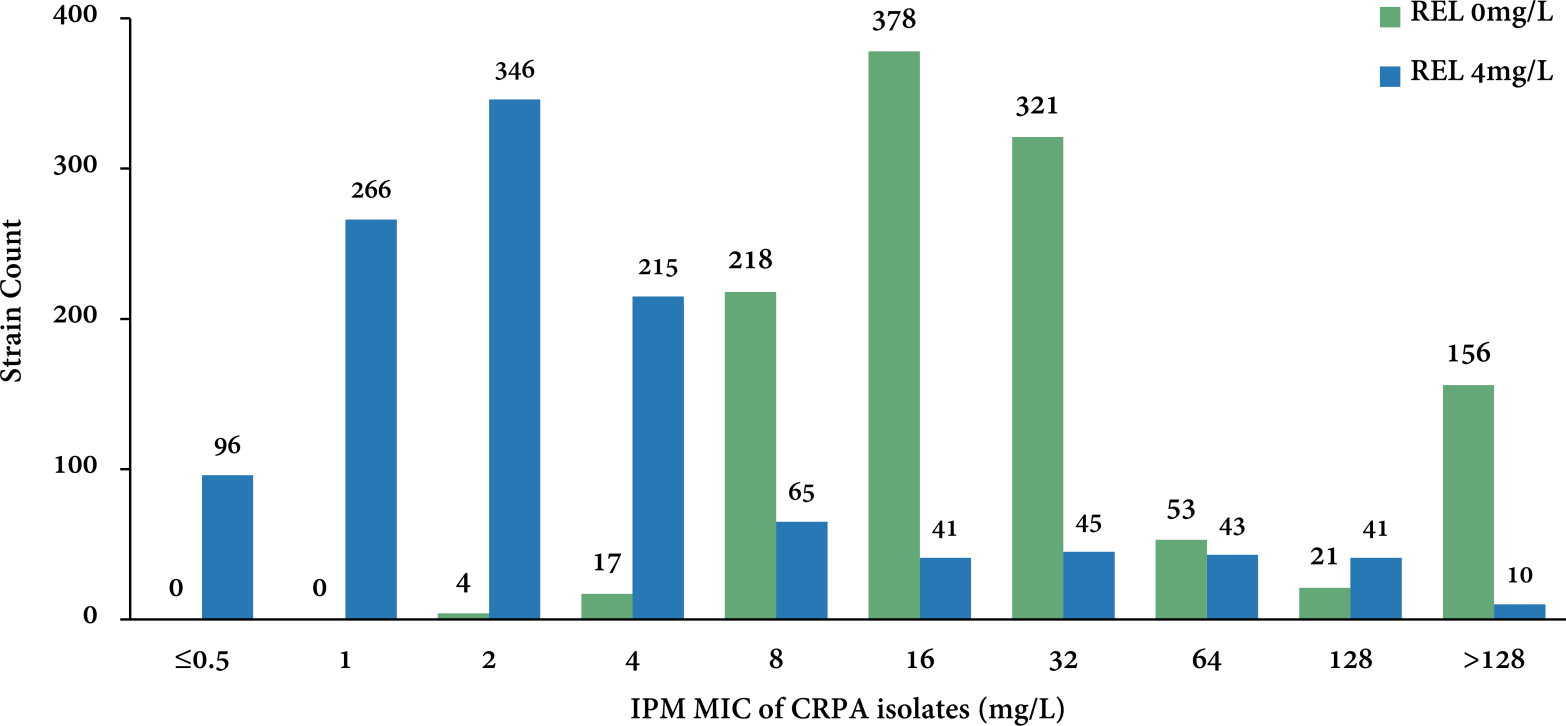
Imipenem MIC distribution of 1,168 CRPA isolates in the absence and presence of 4 mg/L relebactam. CRPA, carbapenem-resistant *Pseudomonas aeruginosa*; IPM, imipenem; MIC, minimum inhibitory concentration; REL, relebactam.

With a fixed relebactam concentration of 4 mg/L, IR MICs were determined and compared with imipenem MICs to explore the antibacterial activity of IR against CRPA isolates. In general, 1,147/1,168 (98.2%) isolates were resistant to imipenem with an MIC_50/90_ of 16/>128 mg/L; however, only 245/1,168 (21.0%) isolates were resistant to IR with an MIC_50/90_ of 2/32 mg/L (Table 1). The addition of 4 mg/L relebactam led to an eightfold decrease in imipenem MIC_50_ value and at least an eightfold decrease in imipenem MIC_90_ value (Table 1; Fig. 1). The overall results demonstrated the notable *in vitro* activity of IR against clinical CRPA isolates.

**TABLE 1:** *In vitro* activities of imipenem and imipenem/relebactam against *Pseudomonas aeruginosa* isolates in this study^*a*^

	MIC Range (mg/L)	MIC_50_ (mg/L)	MIC_90_ (mg/L)	R%	I%	S%
CRPA (*n* = 1,168)						
Imipenem	2 to >128	16	>128	98.2	1.5	0.3
Imipenem/relebactam	0.12/4 to >128/4	2/4	32/4	21.0	18.4	60.6
ST463-PA (*n* = 115)						
Imipenem	16 to >128	>128	>128	100	0	0
Imipenem/relebactam	1/4 to >128/4	64/4	128/4	81.7	10.4	7.8
ST235-PA (*n* = 53)						
Imipenem	8 to 128	32	64	100	0	0
Imipenem/relebactam	2/4 to 128/4	16/4	32/4	52.8	28.3	18.9
ST463 KPC-2-PA (*n* = 104)						
Imipenem	16 to 1024	512	1024	100	0	0
Imipenem/relebactam	1/4 to 256/4	64/4	128/4	90.4	1.0	8.7
	0.5/16 to 64/16	16/16	32/16	80.8	8.7	10.6
ST235 GES-5-PA (*n* = 27)						
Imipenem	32 to 128	64	64	100	0	0
Imipenem/relebactam	16/4 to 128/4	32/4	32/4	100	0	0
	4/16 to 64/16	16/16	16/16	96.3	3.7	0

^*a*^ CRPA, carbapenem-resistant *Pseudomonas aeruginosa*; I%, percentage of intermediate isolates; MIC, minimum inhibitory concentration; R%, percentage of resistant isolates; S%, percentage of susceptible isolates; ST, sequence type.

**Fig 2:**
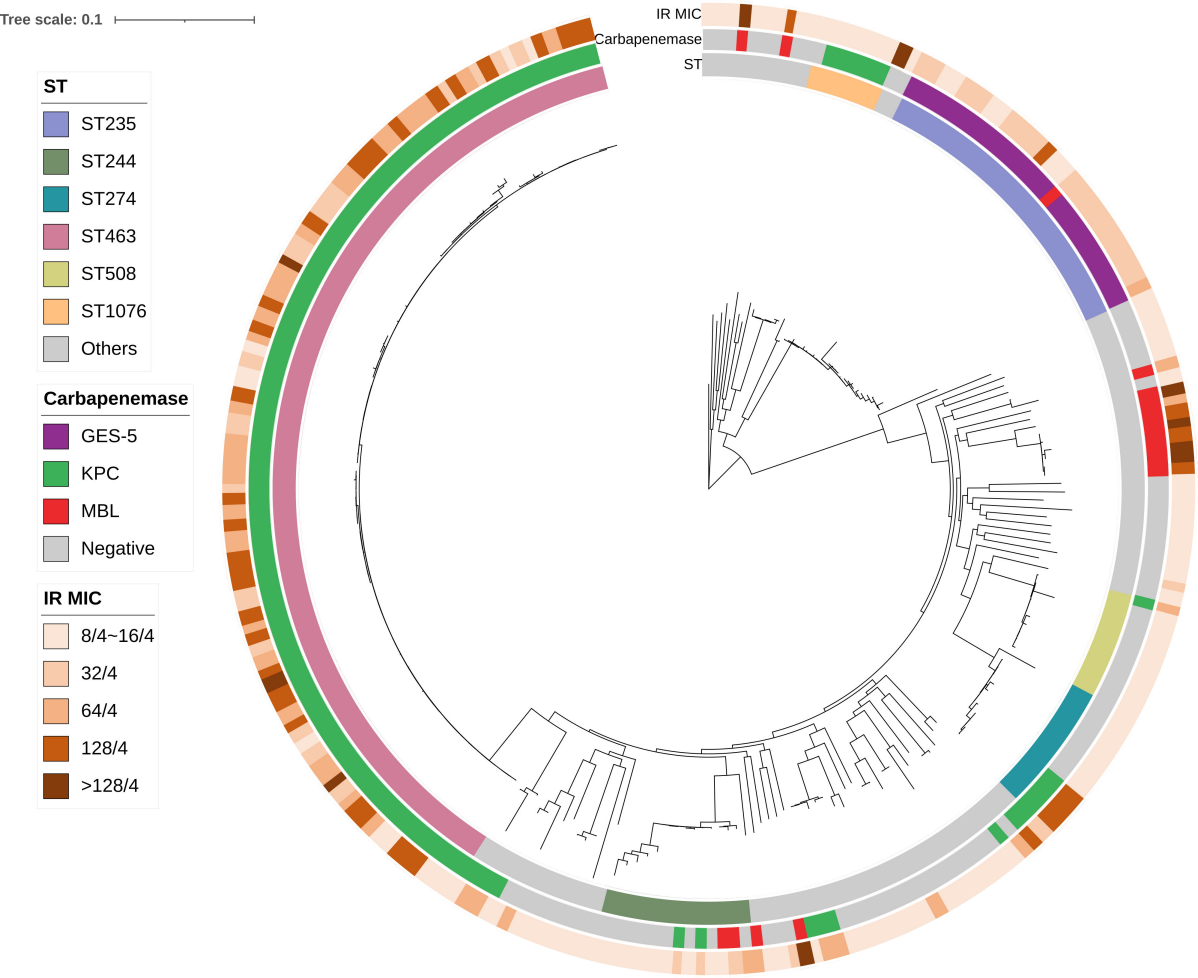
Core genome phylogenetic tree of IR-resistant *Pseudomonas aeruginosa* isolates. Sequence type (innermost ring), carbapenemase carriage (middle ring), and IR MIC (outmost ring) are indicated. IR, imipenem/relebactam; MBL, metallo-β-lactamase; MIC, minimum inhibitory concentration; ST, sequence type.

The phylogenetic tree was then constructed for IR-resistant isolates, and the clonal characteristics were analyzed. Among 245 IR-resistant isolates, 62 different STs were identified (excluding five strains with unknown STs). In 16 of these 62 STs, the IR-resistant strain numbers were more than one (Fig. S3). As shown in Fig. 2, ST463-PA and ST235-PA were still the most prevalent, together accounting for 49.8% (122/245) of total IR-resistant isolates. Notably, differing from the low-level IR resistance observed in most non-ST463 non-ST235 isolates, a significantly higher IR resistance level (MIC 32/4 to >128/4 mg/L) was observed in the majority of ST463 and ST235 isolates, especially when excluding MBL producers (Fig. 2). In terms of the effect of 4 mg/L relebactam, restoration of imipenem susceptibility was only achieved in 7.8% (9/115) of ST463-PA and 18.9% (10/53) of ST235-PA isolates, which was considerably lower than the average (Table 1). Altogether, these resistance profile differences indicated the contribution of clonal characteristics to IR resistance in ST463-PA and ST235-PA.

### Molecular epidemiology and resistance phenotype of ST463-PA and ST235-PA

To further describe the molecular epidemiology and interpret the IR resistance data of ST463 and ST235 isolates, genomic analysis was subsequently conducted. As listed in Table S2, the strong association between class A carbapenemase types and STs could be clearly distinguished. In ST463-PA, 9/115 (7.8%) isolates were non-carbapenemase-producing *P. aeruginosa* (non-CPPA), while 106/115 (92.2%) isolates were KPC-producing *P. aeruginosa* (KPC-PA). The identified KPC alleles included *bla*_KPC-2_ (104/106, 98.1%) and *bla*_KPC-33_ (2/106, 1.9%). In ST235-PA, in addition to 23/53 (43.4%) non-CPPA isolates, 54.7% (29/53) were carbapenemase-type GES-producing *P. aeruginosa* (GES-PA) and 1.9% (1/53) were IMP-15-producing *P. aeruginosa*. The carbapenemase-type GES alleles consisted of *bla*_GES-5_ (27/29, 93.1%) and *bla*_GES-15_ (2/29, 6.9%).

**Fig 3:**
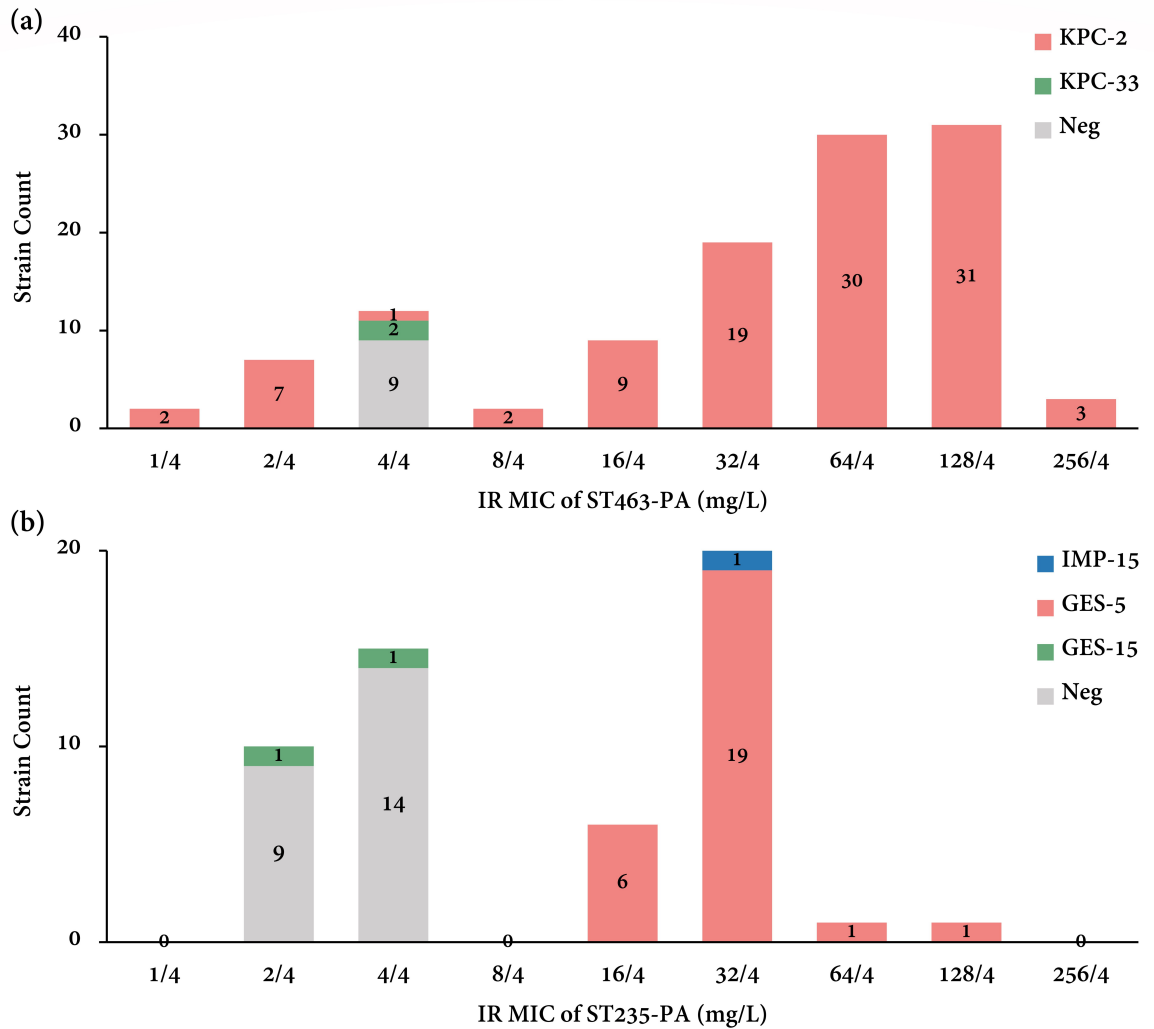
Carbapenemase allele distribution in (**A**) ST463-PA and (**B**) ST235-PA strains exhibiting different IR MICs. IR, imipenem/relebactam; MIC, minimum inhibitory concentration; Neg, negative.

The relationships between carbapenem carriage and IR resistance phenotype in ST463-PA and ST235-PA were also assessed. In brief, ST463 and ST235 non-CPPA isolates were all susceptible or intermediate to IR, but the IR resistance phenotype in ST463 and ST235 CPPA isolates varied. Specifically, in ST463, 2/2 (100%) *bla*_KPC-33_-carrying *P. aeruginosa* were IR intermediate, while 94/104 (90.4%) *bla*_KPC-2_-carrying *P. aeruginosa* were IR resistant (Fig. 3a); in ST235, other than one IMP-15 producer, 2/2 (100%) *bla*_GES-15_-carrying *P. aeruginosa* were IR intermediate or IR susceptible, and 27/27 (100%) *bla*_GES-5_-carrying *P. aeruginosa* were IR resistant (Fig. 3b). Overall, this analysis strongly indicated the potential involvement of KPC-2 and GES-5 in IR resistance in ST463-PA and ST235-PA.

### IR resistance mechanisms in ST463 KPC-2 producers

**Fig 4:**
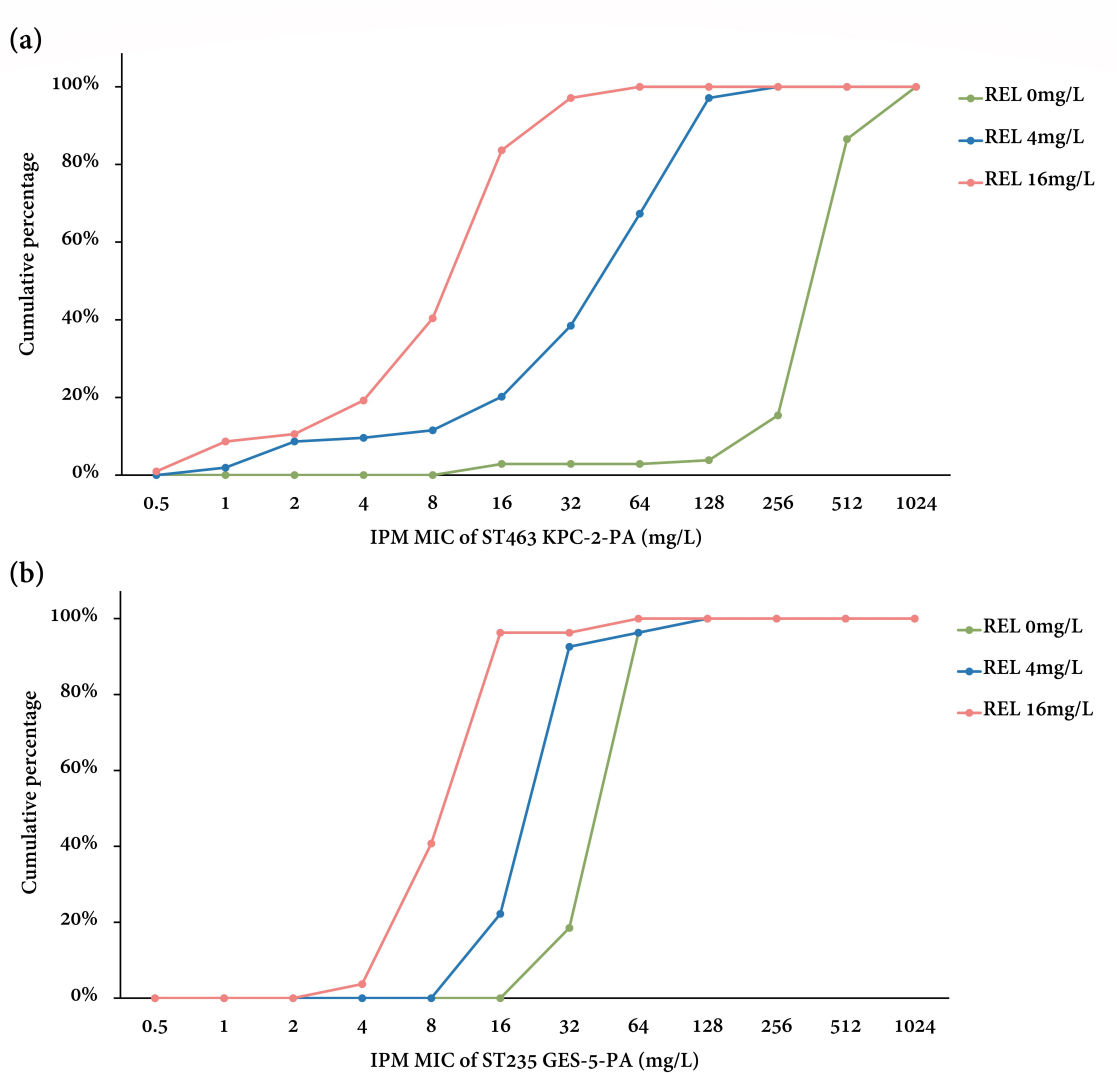
The cumulative percentages of (**A**) ST463 KPC-2-PA and (**B**) ST235 GES-5-PA inhibited at different imipenem concentrations under fixed relebactam concentrations (0 mg/L, 4 mg/L, and 16 mg/L). IPM, imipenem; MIC, minimum inhibitory concentration; REL, relebactam.

IR resistance mechanisms were further explored in ST463 KPC-2 producers. As shown in Fig. 4a and Table 1, 4 mg/L relebactam caused an obvious leftward shift in the cumulative inhibition curves of ST463 KPC-2-PA, resulting in eightfold decreases in both imipenem MIC_50_ and MIC_90_ values. However, with 4 mg/L relebactam, restoration of imipenem susceptibility was only achieved in 9/104 (8.7%) ST463 KPC-2-PA isolates, while the majority still exhibited high-level imipenem resistance (64 mg/L to 256 mg/L). Therefore, we proposed that 4 mg/L relebactam might be insufficient for inhibiting *bla*_KPC-2_ expression in ST463.

To test this hypothesis, first, imipenem MICs were obtained for ST463 KPC-2 producers at a fixed relebactam concentration of 16 mg/L. When administered 16 mg/L relebactam, 20/104 (19.2%) ST463 KPC-2-PA isolates became susceptible or intermediate to imipenem. Notably, with 16 mg/L relebactam, only three ST463 KPC-2 producers still exhibited high-level imipenem resistance (MIC: 64 mg/L). In comparison to 4 mg/L relebactam, additional fourfold reductions in imipenem MIC_50_ and MIC_90_ values were achieved by 16 mg/L relebactam (Table 1).

**Fig 5:**
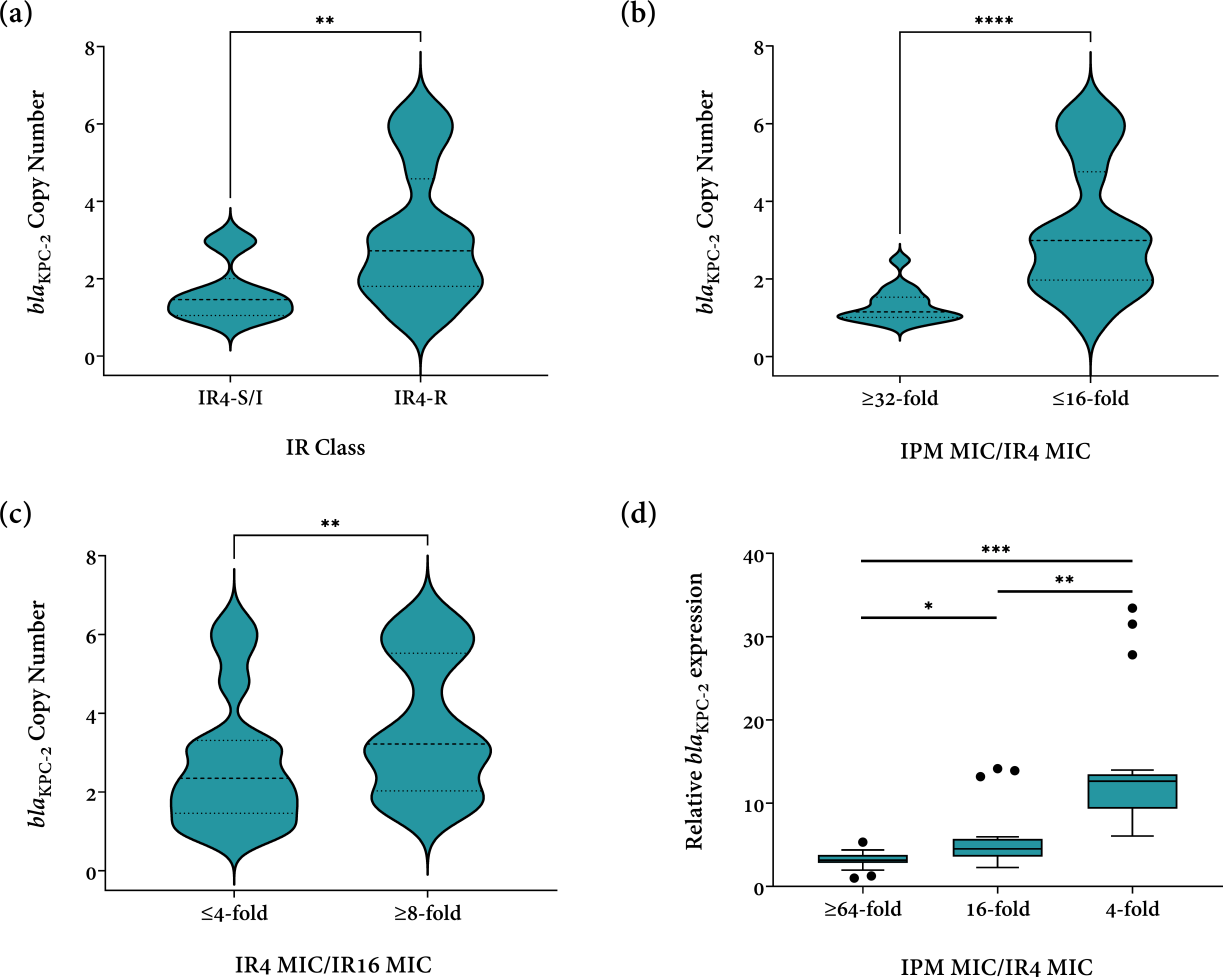
Differences in (**A–C**) *bla*_KPC-2_ copy number and (**D**) relative *bla*_KPC-2_ expression level in ST463 KPC-2-PA strains showing different resistance phenotypes. IPM, imipenem; IR4, imipenem/relebactam (relebactam fixed at 4 mg/L); IR16, imipenem/relebactam (relebactam fixed at 16 mg/L); MIC, minimum inhibitory concentration; R, resistant; S/I, susceptible or intermediate.

Second, *bla*_KPC-2_ copy numbers were compared between ST463 KPC-2 producers showing different IR resistance phenotypes. Compared with ST463 KPC-2-PA isolates susceptible or intermediate to IR, IR-resistant ST463 KPC-2-PA isolates exhibited significantly higher *bla*_KPC-2_ copy numbers (2.7 vs 1.5, *P* < 0.01) (Fig. 5a). In strains showing a ≤ 16-fold imipenem MIC decrease with 4 mg/L relebactam, higher *bla*_KPC-2_ copy numbers were detected (3.0 vs 1.2, *P* < 0.0001) (Fig. 5b). Besides, in strains showing an additional ≥8-fold imipenem MIC decrease with 16 mg/L relebactam, *bla*_KPC-2_ copy numbers were also higher (3.2 vs 2.4, *P* < 0.01) (Fig. 5c). Altogether, these data showed that in ST463 KPC-2-PA, the *bla*_KPC-2_ copy number was associated with not only IR resistance but also the inhibitory effects of relebactam.

Finally, to further verify our hypothesis, the relative *bla*_KPC-2_ expression level was quantitively analyzed. In total, 18 *bla*_KPC-2_-carrying ST463 isolates were selected from three phenotypic groups (≥64-fold, 16-fold, and 4-fold imipenem MIC decrease with 4 mg/L relebactam). These strains shared similar genetic elements surrounding *bla*_KPC-2_ and the same *bla*_KPC-2_ promoter sequences. As shown in Fig. 5d, the relative *bla*_KPC-2_ expression level differed significantly among these three groups (3.1 vs 4.5 vs 12.6, *P* < 0.0001), showing that *bla*_KPC-2_ overexpression was related to the attenuated inhibitory effects of 4 mg/L relebactam.

Although additional decreases in imipenem MICs were achieved when relebactam was increased from 4 mg/L to 16 mg/L, the majority (93/104, 89.4%) of ST463 KPC-2-PA were still not susceptible to imipenem. Previous studies have identified IR resistance associated with mutations in OprD and regulators of MexAB-OprM (19, 20). Thus, we explored the differences in efflux pump overexpression and OprD inactivation between different phenotypic groups in ST463 KPC-2-PA. For efflux pump overexpression, because of the low expression levels of *mexE* and *mexY* genes in ST463 KPC-2-PA, only the expression levels of *mexA* and *mexD* genes were analyzed. Interestingly, under 16 mg/L relebactam, compared with isolates susceptible to imipenem (MIC: 1 mg/L), ST463 KPC-2-PA isolates resistant to imipenem (MIC: 32 mg/L to 64 mg/L) displayed much lower *mexA* and *mexD* expression levels (Fig. S4). This suggested that the IR resistance phenotype differences among ST463 KPC-2-PA could not be attributed to efflux pump overexpression. With regard to OprD inactivation, among imipenem-susceptible and imipenem-nonsusceptible ST463 KPC-2-PA (under 16 mg/L relebactam), the proportions of OprD-inactivating strains were 36.4% (4/11) and 96.8% (90/93), respectively (*P* < 0.0001). This significant difference indicated the potential contribution of OprD inactivation to imipenem resistance, which further led to IR resistance in ST463 KPC-2-PA.

### IR resistance mechanisms in ST235 GES-5 producers

Mechanisms of IR resistance were also investigated in detail for ST235 GES-5-PA. As illustrated in Table 1 and Fig. 4b, neither 4 mg/L nor 16 mg/L relebactam remarkably altered the MIC_50_/MIC_90_ values or the cumulative inhibition curves of imipenem, suggesting that IR resistance in ST235 GES-5-PA could not be attributed to *bla*_GES-5_ overexpression.

Subsequently, the impacts of *bla*_GES_ allelic variants on imipenem and IR resistance were analyzed. The imipenem MICs were generally higher in ST235 GES-5 producers (32 mg/L to 128 mg/L) than in ST235 GES-15 producers (16 mg/L). The IR resistance rate was 100% (27/27) for *bla*_GES-5_-carrying ST235-PA isolates, with MIC_50_/MIC_90_ values of 32/32 mg/L. In contrast, all *bla*_GES-15_-harboring ST235-PA isolates were susceptible or intermediate to IR, exhibiting MIC_50_/MIC_90_ values of 4/4 mg/L. The above data demonstrated the potential involvement of *bla*_GES-5_ carriage in IR resistance.

Cloning experiments were therefore conducted to confirm that IR resistance in ST235 GES-5-PA was mainly attributable to *bla*_GES-5_ carriage (Table 2). Compared to transformants carrying the empty vector, *bla*_GES-5_-carrying transformants displayed 8- to 16-fold increases in imipenem MIC. In *bla*_GES-5_-harboring PAO1 transformants, the addition of 4 mg/L and 16 mg/L relebactam only caused a slight decrease in imipenem MIC. These results indicated that relebactam exerted poor inhibitory effects on GES-5 carbapenemase activity in *P. aeruginosa*, thus resulting in IR resistance in ST235 GES-5-PA.

**TABLE 2:** MICs of β-lactams and BLBLIs for transformants and related clinical strains used in this study^*e*^

	MICs (mg/L) determined by broth microdilution methods								
	AZT	FEP	PTZ^*a*^	CAZ	IPM	IR^*b*^	IR^*c*^	MEM	MV^*d*^
R16-15 (*bla*_GES-5_ positive)	32	16	128	8	32	16	8	64	64
R20-99 (*bla*_GES-15_ positive)	32	64	>128	128	16	2	2	8	4
DH5α pGK1900	0.06	0.06	0.5	0.25	0.12	0.12	0.12	0.03	0.03
DH5α pGK1900_GES-5	0.25	0.25	32	4	2	0.25	0.25	0.25	0.03
DH5α pGK1900_GES-15	2	2	2	128	0.25	0.25	0.12	0.03	0.03
PAO1 pGK1900	8	4	4	4	1	0.25	0.25	0.5	1
PAO1 pGK1900_GES-5	8	32	256	64	8	4	2	128	128
PAO1 pGK1900_GES-15	128	>128	64	>128	2	0.5	0.5	2	2

^*a*^ Tazobactam was added at a fixed concentration of 4 mg/L.

^*b*^ Relebactam was added at a fixed concentration of 4 mg/L.

^*c*^ Relebactam was added at a fixed concentration of 16 mg/L.

^*d*^ Vaborbactam was added at a fixed concentration of 8 mg/L.

^*e*^ AZT, aztreonam; BLBLI, β-lactam-β-lactamase inhibitor combination; CAZ, ceftazidime; FEP, cefepime; IPM, imipenem; IR, imipenem/relebactam; MEM, meropenem; MIC, minimum inhibitory concentration; MV, meropenem/vaborbactam; PTZ, piperacillin/tazobactam.

## DISCUSSION

Given the increasing clinical availability of IR and the severe threat of *P. aeruginosa* high-risk clones, an understanding of IR resistance in *P. aeruginosa* high-risk clones is urgently needed. In this study, we explored the IR resistance profiles of 1,168 clinical CRPA isolates from China, which revealed high-risk clones ST463 and ST235 as the major contributors to IR resistance. ST463 and ST235 clones in this study were characterized by KPC and GES carbapenemase carriage, respectively. Further experiments and genomic analysis demonstrated that *bla*_KPC-2_ overexpression combined with OprD inactivation mediated high-level IR resistance in ST463-PA, while *bla*_GES-5_ carriage mediated relatively low-level IR resistance in ST235-PA.

Several studies have reported the *in vitro* or *in vivo* dynamic evolution of IR resistance in *P. aeruginosa*, mainly focusing on the contribution of the mutational resistome. In a stepwise resistance development study (20), Gomis-Font et al*.* obtained IR-resistant mutants of wild-type and hypermutable PAO1 strains by serial passage. Most mutants only exhibited low-level IR resistance, which was associated with OprD inactivation and MexAB-OprM overexpression. High-level IR resistance could be achieved if an additional mutation (PBP1a T680A) was acquired. In another *in vitro* study (19), Gomis-Font et al*.* conducted the same experiments on three *P. aeruginosa* high-risk clones. Similarly, only borderline resistance was identified, which was caused by MexB or ParS structural mutations. Recently, Shields et al*.* reported the emergence of IR nonsusceptibility following the treatment of MDR-PA infections for the first time (21). Out of 19 patients treated with IR, isolates not susceptible to IR were recovered from five patients. All isolates displayed moderate IR resistance related to MexAB-OprM and/or MexEF-OprN mutation, which could be reverted by PAβN inhibition. Overall, the promising antibacterial effects of IR in non-CPPA could be concluded.

Previous biochemical analysis has demonstrated the stability of the KPC-2-relebactam complex, probably due to the lack of essential water molecules near relebactam sulfate (22). This is consistent with the published antimicrobial susceptibility testing results. In KPC-producing non-Proteeae *Enterobacteriaceae*, the IR susceptibility rates were generally very high, ranging from 96.3% to 100% (22 – 26). In *P. aeruginosa*, although only two studies included KPC-producing isolates, they both reported 100% susceptibility rates (27, 28).

Generally, IR resistance related to KPC enzymes has seldom been reported. Until 2022, four studies investigating IR resistance development in KPC-producing *K. pneumoniae* (KPC-KP) were published. In one *in vitro* multistep selection study (29), IR-resistant mutants were selected for five KPC-KP strains. WGS and quantitative PCR confirmed OmpK36 inactivation, KPC allele mutations, and increased *bla*_KPC_ copy number as the major IR resistance mechanisms. In other studies describing the *in vivo* evolution of IR resistance (30 – 32), Gaibani et al*.* isolated three IR-resistant KPC-KP strains, which were compared with the corresponding parental KPC-KP strains susceptible to IR. An increased *bla*_KPC_ copy number was confirmed in all IR-resistant strains, manifesting the rapid resistance evolution in KPC producers. Similar to the findings described above, we demonstrated that increased *bla*_KPC-2_ copy number and *bla*_KPC-2_ expression level were also associated with IR resistance in *P. aeruginosa*. Concerningly, ST463 KPC-2-PA isolates in our study were never exposed to IR before collection, but they still showed an extremely high IR resistance rate (90.4%). Under these circumstances, clinical treatment choices would be greatly constrained.

Although GES producers were found among IR-resistant *P. aeruginosa* in previous studies (27, 33), the causal relationship between GES and IR resistance is uncertain. With GES-15 as the reference, the low-level IR resistance mediated by GES-5 in *P. aeruginosa* was confirmed by cloning experiments in this study. This is consistent with the enzymatic properties of GES-20 (identical to GES-5 with the leader sequence removed). According to Hujer et al.’s study (34), GES-20 possesses a constrained Ω loop; thus, relebactam is positioned in an unstable conformation, which impairs the inhibitory effect of relebactam.

The poor outcome associated with ST235 and ST463 CRPA, mainly caused by their hypervirulence and MDR/XDR/PDR profiles, is concerning. Generally, hypervirulence is defined as the ability to cause higher mortality and more severe acute infections (1). ST235 CRPA isolates typically possess a hypervirulent *exoU*+ genotype and various resistance-related mutations. In a study on *P. aeruginosa* bacteremic pneumonia, a 29.8% increase in 5-day mortality and a 34.4% increase in 30-day mortality were noted in patients infected with ST235-PA (4). Similarly, harboring both *exoU* and *exoS* genes, the ST463 clone also exhibits a hypervirulent phenotype. In a recent study by Zhang et al*.* (35), the virulence level of different strains was assessed by cytotoxicity assay and the *Galleria mellonella* larvae infection model. The included ST463-PA isolates showed hypervirulence comparable to that of ST235-PA. Moreover, in a clinical retrospective cohort study conducted in East China (5), ST463 CRPA accounted for 48.0% of total CRPA BSI cases and 69.6% of total death cases, highlighting its serious threat. Thus, therapeutic options should be carefully considered to improve clinical outcomes in patients infected with ST235-PA and ST463-PA.

In conclusion, our data highlighted the distinct IR resistance characteristics between CRPA isolates from high-risk clones and other STs. Of particular concern is the association of high-risk clones with IR resistance phenotype and XDR/DTR phenotype, which may worsen clinical outcomes and restrict therapeutic options. Using whole-genome analysis methods and quantitative PCR, we identified *bla*_KPC-2_ overexpression as the major driver of high-level IR resistance in ST463-PA. In addition, we also confirmed that *bla*_GES-5_ carriage mediated IR resistance in ST235-PA. Overall, this epidemiological survey systematically deciphered the IR resistance mechanisms in *P. aeruginosa* global high-risk clone ST235 and regional high-risk clone ST463.

## Data Availability

The nucleotide sequences analyzed in this study have been deposited at DDBJ/ENA/GenBank under the accession numbers JASAFP000000000–JASAMA000000000.

## SUPPLEMENTAL MATERIAL

Supplemental figures and tables aac.00675-23-s0001.docx Tables S1 and S2 and Fig. S1 to S4.

ASM does not own the copyrights to Supplemental Material that may be linked to, or accessed through, an article. The authors have granted ASM a non-exclusive, world-wide license to publish the Supplemental Material files. Please contact the corresponding author directly for reuse.
