# Supplementary material for: bla KPC-2 overexpression and bla GES-5 carriage as major imipenem/relebactam resistance mechanisms in Pseudomonas aeruginosa high-risk clones ST463 and ST235, respectively, in China
Source: Antimicrob Agents Chemother. 2023 Oct 11;67(11):e00675-23. doi: 10.1128/aac.00675-23 (PMC10649045; doi:10.1128/aac.00675-23)
Supplement: Supplemental figures and tables — Tables S1 and S2 and Fig. S1 to S4. [file aac.00675-23-s0001.docx]

**Supplementary Materials**

***bla*_KPC-2_ overexpression and *bla*_GES-5_ carriage as major imipenem/relebactam resistance mechanisms in *Pseudomonas aeruginosa* high-risk clones ST463 and ST235, respectively, in China**

**Running Title:** KPC-2 and GES-5 cause imipenem/relebactam resistance

Yue Li^1,2,3#^, Li Fang^1,2,3#^, Mengqian Dong^1,2,3^, Heng Cai^1,2,3^, Xiaoting Hua^1,2,3^, Yan Jiang^1,2,3^, Yunsong Yu^1,2,3*^, Qing Yang^4,5*^

^1^Department of Infectious Diseases, Sir Run Run Shaw Hospital, Zhejiang University School of Medicine, Hangzhou, China.

^2^Key Laboratory of Microbial Technology and Bioinformatics of Zhejiang Province, Hangzhou, China.

^3^Regional Medical Center for National Institute of Respiratory Diseases, Sir Run Run Shaw Hospital, Zhejiang University School of Medicine, Hangzhou, China.

^4^Department of Laboratory Medicine, The First Affiliated Hospital, Zhejiang University School of Medicine, Hangzhou, China.

^5^State Key Laboratory for Diagnosis and Treatment of Infectious Diseases, National Clinical Research Center for Infectious Diseases, Collaborative Innovation Center for Diagnosis and Treatment of Infectious Diseases, The First Affiliated Hospital, Zhejiang University School of Medicine, Hangzhou, China.

^#^These authors contributed equally to this work.

^*^Correspondence:

Qing Yang

address: The First Affiliated Hospital, Zhejiang University School of Medicine, Hangzhou, China, 310003

email: [yq721227@zju.edu.cn](mailto:yq721227@zju.edu.cn)

tel: +86-571-87237285

Yunsong Yu

address: Sir Run Run Shaw Hospital, Zhejiang University School of Medicine, Hangzhou, China, 310016

email: [yvys119@zju.edu.cn](mailto:yvys119@zju.edu.cn)

tel: +86-571-86006660

**Table S1.** **Primers and conditions used in this study**

| Target Gene | Primers (5'-3') | Tm |
| --- | --- | --- |
| Quantitative PCR |  |  |
| *bla*_KPC-2_ gene | **F:** GATGCGCGCGATACCTCATC | 60℃ |
|  | **R:** AGTGCAGAGCCCAGTGTCAG |  |
| *rpoD* gene | **F:** CTTACGCGGAGGTCAACGAC | 60℃ |
|  | **R:** TCCGGGGCTGTCTCGAATAC |  |
| *mexA* gene | **F:** GGCGACAACGCGGCGAAGG | 60℃ |
|  | **R:** CCTTCTGCTTGACGCCTTCCTGC |  |
| *mexD* gene | **F:** CGAGCGCTATTCGCTGC | 60℃ |
|  | **R:** GGCAGTTGCACGTCGA |  |
| *mexE* gene | **F:** TCATCCCACTTCTCCTGGCGCTACC | 60℃ |
|  | **R:** CGTCCCACTCGTTCAGCGGTTGTTCGATG |  |
| *mexY* gene | **F:** CCGCTACAACGGCTATCCCT | 60℃ |
|  | **R:** AGCGGGATCGACCAGCTTTC |  |
| Cloning experiment |  |  |
| *bla*_GES_ gene and  promoter region | **F:** CGACTCACTATAGGGCGAACCGTTCCAT ACAGAAGCTG | 53℃ |
|  | **R:** GAGGGCAGAGCCATGAGAAAACTATTTG TCCGTGCTCAG |  |
| pGK1900 plasmid  backbone | **F:** TTCTCATGGCTCTGCCCTC | 56℃ |
|  | **R:** TTCGCCCTATAGTGAGTCGT |  |

**Table S2.** **Carbapenemase type distribution and its association with IR resistance phenotype in ST463 and ST235 *Pseudomonas aeruginosa*.**

| Sequence  Type | Carbapenemase  Type | IR  Phenotype | Strain  Number |
| --- | --- | --- | --- |
| ST463 (*n*=115) | KPC-2 | R | 94 |
|  | KPC-2 | S/I | 10 |
|  | KPC-33 | S/I | 2 |
|  | Negative | S/I | 9 |
|  |  |  |  |
| ST235 (*n*=53) | GES-5 | R | 27 |
|  | GES-15 | S/I | 2 |
|  | IMP-15 | R | 1 |
|  | Negative | S/I | 23 |

IR, imipenem/relebactam; R, resistant; S/I, susceptible or intermediate; ST, sequence type.

**
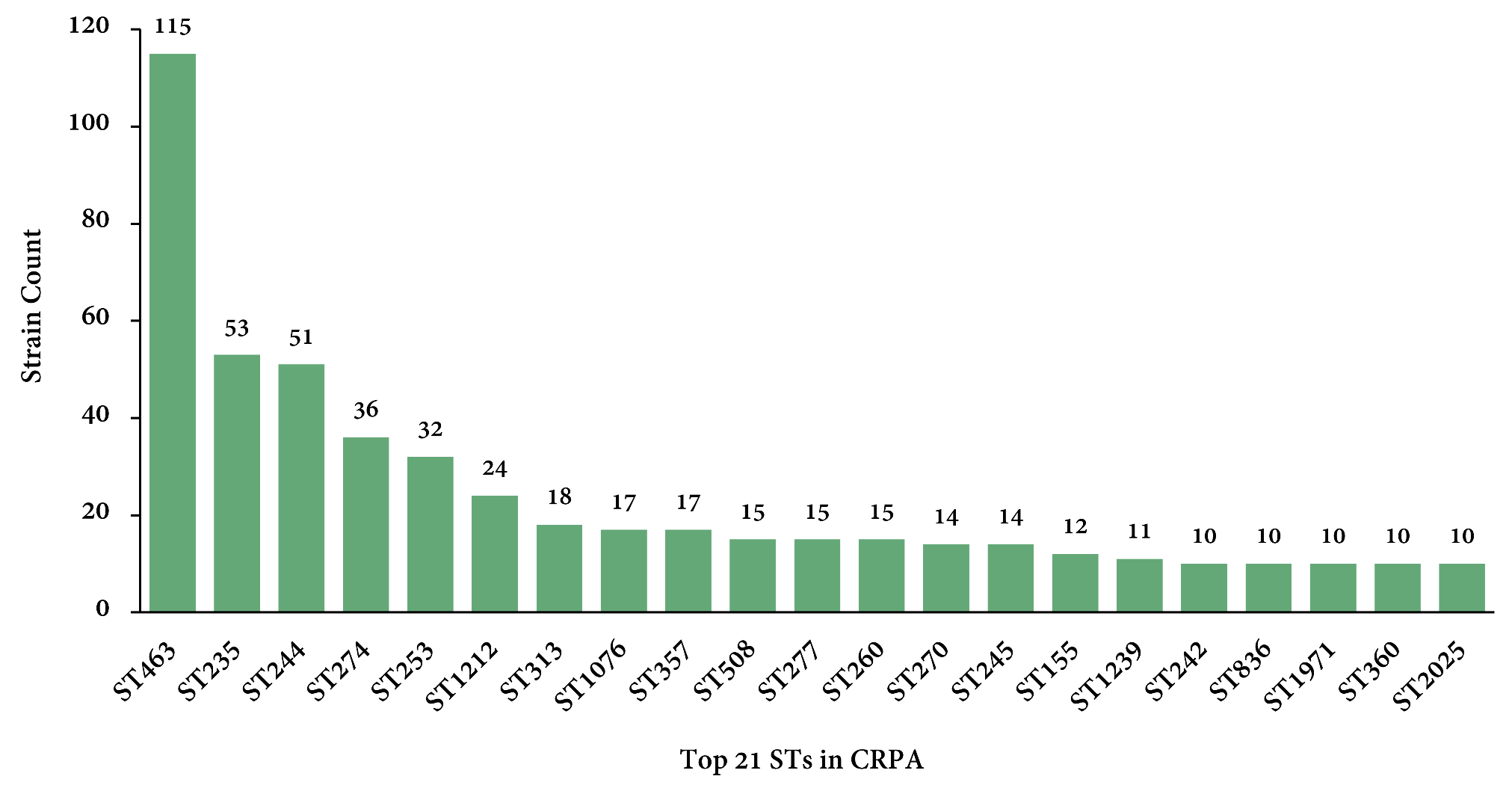
**

**Figure S1. ST distribution of 1,168 CRPA isolates in this study.** Only STs with more than nine strains are displayed. CRPA, carbapenem-resistant *Pseudomonas aeruginosa*; ST, sequence type.

**
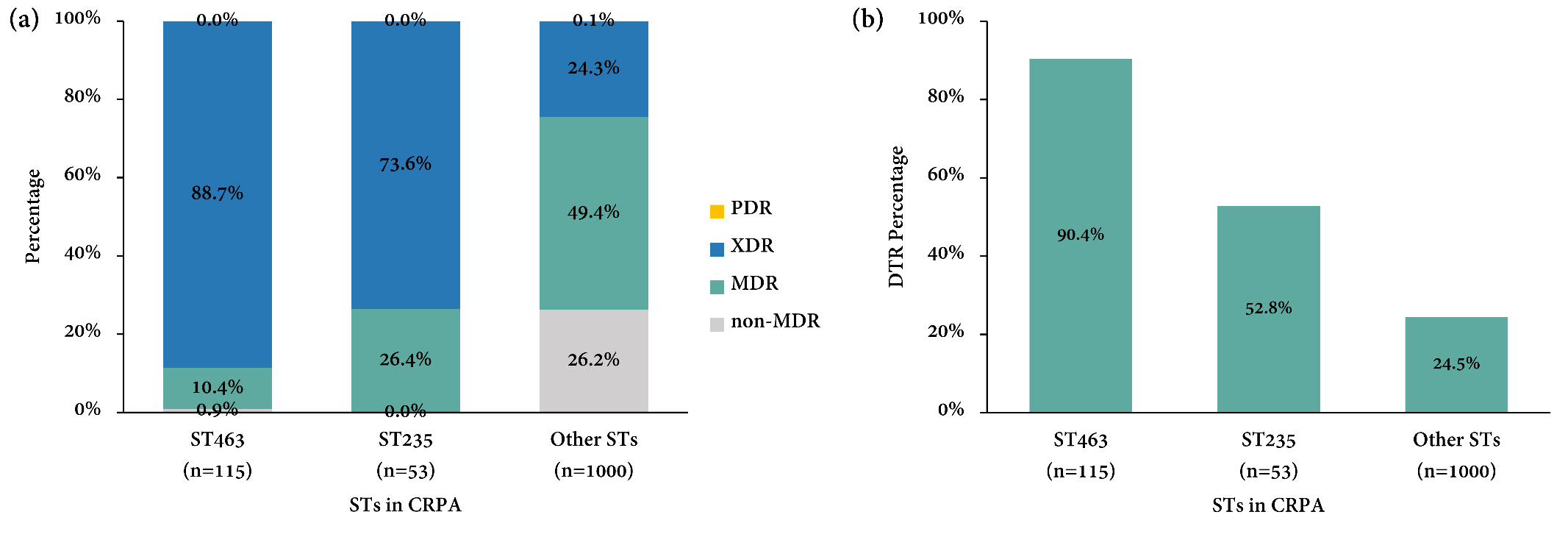
**

**Figure S2. The proportion of isolates with (a) XDR phenotype and (b) DTR phenotype in CRPA from different STs.** CRPA, carbapenem-resistant *Pseudomonas aeruginosa*; DTR, difficult-to-treat resistant; MDR, multidrug-resistant; PDR, pandrug-resistant; ST, sequence type; XDR, extensively drug-resistant.


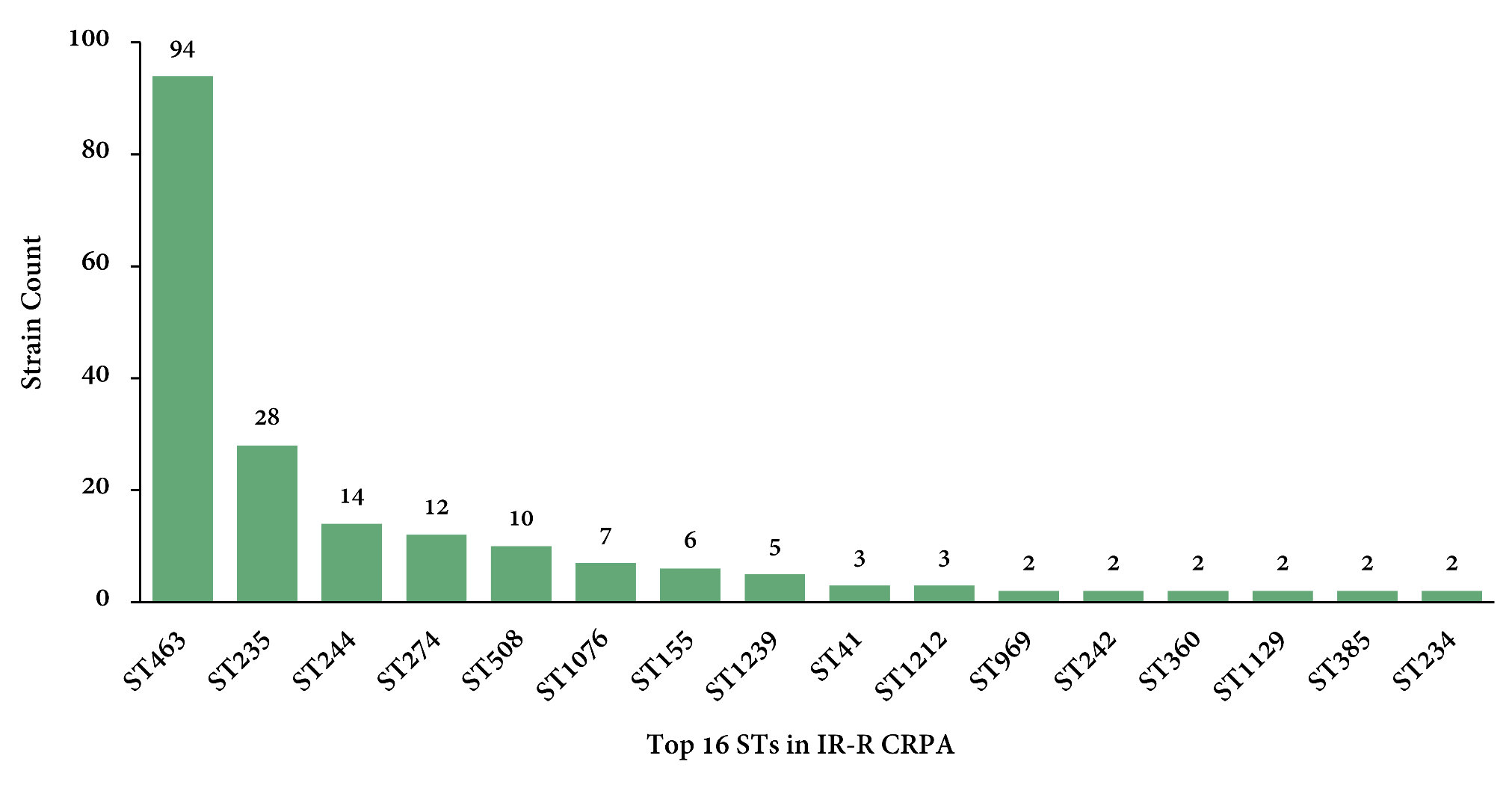


**Figure S3. ST distribution of 245 IR-resistant CRPA isolates in this study.** Only STs with more than one strain are displayed. CRPA, carbapenem-resistant *Pseudomonas aeruginosa*; IR-R, imipenem/relebactam-resistant; ST, sequence type.

**
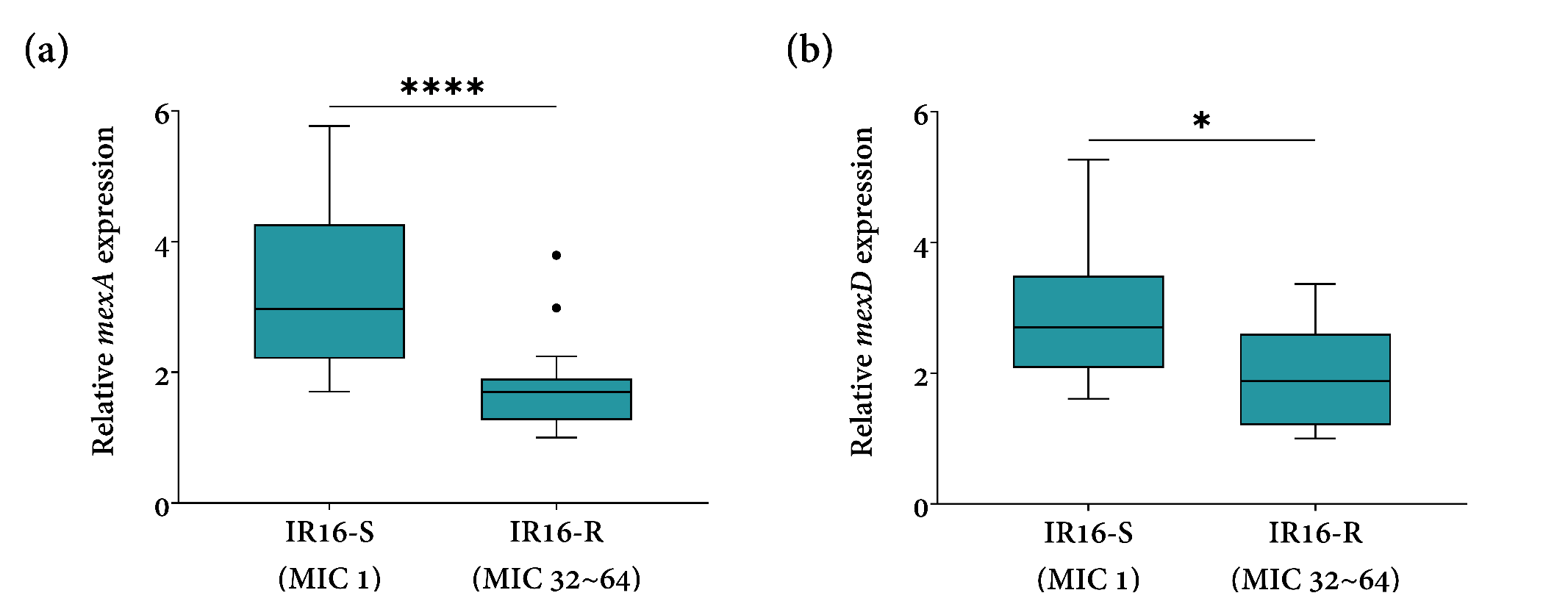
**

**Figure S4.** **Differences in relative (a) *mexA* and (b) *mexD* expression levels in ST463 KPC-2-producing *Pseudomonas aeruginosa* showing different resistance phenotypes.** IR16, imipenem/relebactam (relebactam fixed at 16 mg/L); MIC, minimum inhibitory concentration; R, resistant; S, susceptible.
